# A method for generating uniform size-segregated pyrite particle fractions

**DOI:** 10.1186/1467-4866-8-9

**Published:** 2007-10-10

**Authors:** Amy L Wolfe, Ran Liu, Brian W Stewart, Rosemary C Capo, David A Dzombak

**Affiliations:** 1 Department of Geology and Planetary Science, University of Pittsburgh, Pittsburgh, PA 15260, USA; 2 Department of Civil and Environmental Engineering, Carnegie Mellon University, Pittsburgh, PA 15213, USA; 3 ARRO Consulting, Inc., 270 Granite Run Drive, Lancaster, PA 17601, USA

## Abstract

**Background:**

Standardized sample preparation techniques allow comparison of pyrite dissolution experiments under diverse conditions. Our objective was to assess dry and wet sieving preparation methodologies, and to develop a reproducible technique that yields uniformly size-distributed material within a limited size range of interest.

**Results:**

Here, we describe a wet sieving preparation method that successfully concentrates pyrite particles within a 44–75 μm diameter range. In addition, this technique does not require a post-processing cleanup step to remove adhering particles, as those particles are removed during the procedure. We show that sample preparation methods not only affect the pyrite size distribution, but also apparent dissolution rates.

**Conclusion:**

The presented methodology is non-destructive to the sample, uses readily available chemical equipment within the laboratory, and could be applied to minerals other than pyrite.

## Background

Pyrite, FeS_2_, is one of the most abundant sulfide minerals at the Earth's surface and represents an important reservoir for iron and sulfur within the Earth's crust. It exists in a variety of forms and is prevalent in numerous environments including hydrothermal ore zones, modern lake and ocean sediments, and sedimentary rocks [1-6]. Regardless of its source, the weathering of pyrite via oxidative dissolution can result in the acidification and degradation of water resources [[6-13] and references therein]. The rates and mechanisms governing this process are only partially understood despite numerous experimental studies of pyrite oxidation [14-22].

In pyrite dissolution and oxidation experiments, massive hydrothermal pyrite is normally used because it is readily available and well characterized. However, sedimentary pyrite exists in many forms, and pyrite mineral preparation methodologies are inconsistent within the literature (Table 1). Previous research [14,15] indicates that differences in grain size (i.e., surface area) can exert significant control on pyrite oxidation rates, and, in general, there is a positive, linear correlation between surface area and the rate of pyrite oxidation [23]. Pyrite powders are usually prepared by grinding a homogenous, massive pyrite sample using an agate mortar and pestle [10,16-18] or a mixer mill [24]. To achieve a specific size range of material for experiments, samples are either dry sieved [17,18], wet sieved [16], or both [11]. Sieved samples are then cleaned in various ways to remove fine particles adhering to the mineral surface and oxidation products prior to use.

**Table 1:** Pyrite preparation methodologies used in previous studies. In each method listed, the pyrite was hydrothermal in origin.

**Target Size Fraction (μm)**	**Methodology**	**Reference**
125 – 250 μm	The pyrite was crushed, soaked overnight in hot hydrofluoric acid, washed in distilled water, dried in air and sieved. Sieved pyrite was ultrasonically cleaned in ethanol, rinsed with 1 M nitric acid for one minute, triply rinsed with distilled water, and then with ethanol. The pyrite was dried with air and stored briefly in beakers.	[14] McKibben, M.A., and Barnes, H.L. (1986)
40 – 80 μm	Powders were prepared by grinding in an agate mortar. The oxidation products were eliminated by rinsing with 10^-2 ^MHNO3.	[15] Bonnisell – Gissinger, P. et al. (1998)
74–177 μm	Samples were crushed using an agate mortar and pestle. The crushed pyrite was soaked overnight in hot hydrofluoric acid, washed in deoxygenated deionized water, dried in air, and sieved.	[10] Kamei, G., and Ohmoto, H. (2000)
105 – 150 μm	Samples were dry ground in two steps: 1) a glass-cleaned ring pulverizer was used to reduce grain size and 2) an agate mortar was used to crush the particles to the desired particle size range. The ground pyrite was dry sieved. Samples were kept in a glass desiccator under vacuum after preparation to avoid surface oxidation.	[16] Cruz, R., et al. (2001)
150–250 μm	Pyrite was ground using an agate mortar, sieved with ethanol, and then washed in an ultrasonic bath. Procedure was repeated until the ethanol was clear and free of fine particles after the ultrasonic bath treatment.	[20] Descostes, M., et al. (2004)
150 – 500 μm	Crushed minerals were sieved, ultrasonically treated and washed repeatedly to remove fine particles, and then treated with 10% HCl for 2 hours to remove any preexisting oxide layer. The crushed mineral particles were rinsed with ethanol and allowed to dry.	[17] McGuire, M.M., et al. (2001)
-0.30 mm	Material was classified into various size fractions by wet-dry screening. Prior to leaching experiments, samples of the ground material were soaked in 3 M hydrochloric acid solution for 36 h, filtered, rinsed with double-distilled water, dried with acetone, and kept under vacuum in a desiccator.	[18] Caldeira, C.L. et al. (2003)
250 – 420 μm	The pyrite was crushed, sieved, and rinsed with ethyl alcohol three to five times until the supernatant was clear. The samples were then sonicated in ethyl alcohol (repeated at least three times until the supernatant was clear). The grains were dried at 70°C for 12 h.	[19] Jerz, J.K., and Rimstidt, D. (2004)
37 – 74 μm	Pyrite was ground in air for different periods. After grinding, samples were sieved under dry conditions and the size fraction between 200 and 400 mesh collected.	[24] Sasaki, K. (1994)

The objective of this work was to develop an effective, reproducible procedure for isolating pyrite grains in the 44–75 μm range for dissolution studies. This work was conducted as part of a comparative investigation of dissolution rates for pyrite from different petrogenetic environments. Previous pyrite dissolution experiments (Table 1) involved hydrothermal pyrite particles >75 μm in diameter, while our experiments called for a smaller size fraction, 44–75 μm, to better simulate dissolution of finely disseminated pyrite in some sedimentary environments. We compared dry and wet sieving preparation methodologies with the goal of developing a reproducible technique that yields clean material within our size range of interest. The methods were evaluated through a combination of SEM analysis and batch dissolution experiments.

## Methods

### Crushing and Sieving Procedures

#### Sample Crushing

Five pyrite samples, two hydrothermal and three sedimentary in origin, were used to compare the effectiveness of dry and wet sieving techniques. The starting samples were either massive euhedral or nodular (Table 2). Nodular samples were cut using a trim saw. For square and rectangular samples, the outside edges were removed to obtain a pristine internal sample. Spherical samples were cut into smaller square/rectangular pieces and the outside surface was removed using 220-mesh silicon carbide grit. Samples of 30–50 g were collected and crushed into pea-size pieces using a sledgehammer. The sledgehammer, steel plate and sample were wrapped in aluminum foil to prevent contamination.

**Table 2:** Samples used in this study.

**Sample ID**	**Source**	**Morphology**	**Petrogenetic Environment**	**Mineralogy**	**Molar S/Fe**
HY-001	Wards Natural Science	Euhedral cube	hydrothermal	pyrite	2.01
HY-002	Rock Currier, personal communication	Euhedral cube	hydrothermal	pyrite	2.02
SED-001	Lower Kittanning coal, OH	nodular	sedimentary, within coal	pyrite	2.02
SED-002	Texas	nodular	sedimentary, within coal	pyrite	1.97
SED-003	Calvert Bluff Formation, Texas	spherical nodule	sedimentary, within coal	pyrite with minor quartz	1.97

Composition was determined using x-ray diffraction and molar S:Fe ratio for each sample. All samples had a molar S:Fe ratio of 2:1, indicating insignificant contribution from other species.

#### Powder Preparation and Characterization

Powder preparation techniques using both a mixer mill and a mortar and pestle were evaluated. Most samples of the crushed pyrite (~10 g) were milled into a powder using a tungsten carbide mixer mill for approximately 3 minutes. An aliquot of one sample (HY-001) was also ground in an agate mortar and pestle as a comparison to the mixer mill. Samples were placed in a desiccator under vacuum immediately after being powdered. X-ray diffraction analysis (Philips XRD PW3710; Almelo, Netherlands) indicates that all samples are pyrite, although SED-003 contains minor (≤ 10%) quartz. Additional aliquots (~0.1 g) of pyrite powder were completely dissolved in 10 mL concentrated nitric acid and further diluted to 5% nitric acid for iron and sulfur analysis by ICP-AES. The results (Table 2) indicate that the samples consist of stoichiometric FeS_2_.

#### Dry Sieving

Prior to sieving, the sample was dried in an oven for approximately 30 minutes at 105°C to drive off adsorbed moisture. Given the results of previous pyrite oxidation experiments, the appearance of oxidation products on pyrite surfaces is most likely minor, given the short period of time in which the samples were in the oven [19,25]. In addition, the samples were treated prior to dissolution experiments to ensure the removal of any possible oxidation products (see section on dissolution experiment procedures). Approximately 5–6 grams of material were transferred to a polypropylene sieve set equipped with nylon mesh (41 μm, 63 μm and 75 μm mesh sizes were used) and shaken for 10 minutes in a sieve shaker. Following the dry sieving procedure, a coating of pyrite grains much finer than the smallest sieve size remained on mineral surfaces. To address this issue, two surface cleaning procedures were evaluated: (1) About 10 grams of sieved pyrite were added to ~200 ml tetrabromoethane (density = 2.89 g/cm^3^) within a 250 ml separatory funnel. After 20 minutes, the settled pyrite was collected from the bottom of the column and cleaned with acetone. (2) Two to three grams of sieved pyrite grains were placed into a 50 ml polypropylene test tube. Approximately 35 ml of 70% ethanol were added to each tube and the sample was ultrasonicated for 1 minute. Suspended material within the solution was decanted and discarded. Following both procedures, the remaining samples were oven-dried 30–60 minutes at 105°C, then transferred to a desiccator and placed under vacuum. Sub-samples were collected for SEM analysis to determine the size range of the particles collected.

#### Wet Sieving

**Figure 1:**
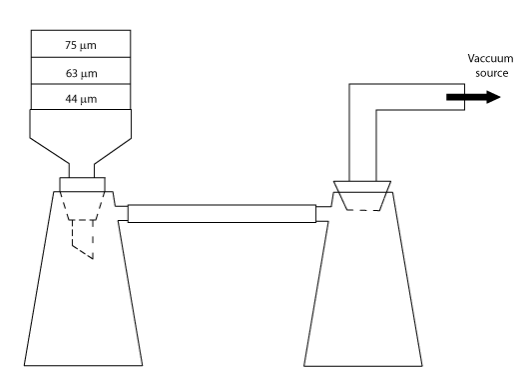
**Wet sieving apparatus**. Size fractions are collected using an adapted vacuum filtration technique. Water and ethanol are collected in the left flask. Sieves used in these experiments were 70 mm in diameter.

A vacuum filtration technique was used to obtain multiple fractions of pyrite. In initial experiments, we captured the 63–75 μm size fraction; however, we found that this size range did not provide enough material for our dissolution experiments. The size range was then broadened to capture pyrite particles 44–75 μm in diameter. Three-inch brass sieves, mesh sizes 200 (74 μm), 230 (63 μm) and 325 (45 μm) were inserted tightly within a one-piece porcelain Büchner funnel with a fixed perforated filter (Fig. 1). Whatman No. 54 filter paper at the base of the Büchner funnel was used to trap material finer than 20 microns. A rubber crucible adapter was used to ensure a tight seal between the funnel and 500 ml Pyrex side arm flask. Tygon tubing (3/8 × 1/8 inch) was used to connect this set-up to a water trap. The water trap consisted of another 500 ml side arm flask connected to Tygon tubing using a 6.5 rubber stopper with a removable glass stem. Each of the Pyrex side arm flasks were attached to support stands using adjustable angle clamps. The water trap was connected to a vacuum pump, which was necessary for sufficiently rapid sieving.

To begin the procedure, powdered material was transferred into 50-ml polypropylene test tubes. Ethanol (35 ml of 70%) was then added to each tube and the sample was ultrasonicated for 1 minute. The material was then poured onto the top of the sieve stack to begin the wet sieving process. Alternating aliquots of ultrapure water and 50% ethanol (ending with ethanol) were added until the entire sample had been sieved. Ethanol was used to prevent pyrite oxidation during sieving. Finally, the remaining samples were oven-dried 30–60 minutes at 105°C, then transferred to a desiccator and placed under vacuum. Sub-samples were collected for SEM analysis to determine the size range of the particles collected.

Samples SED-001 and SED-002, both of which were extracted from a coal matrix, appeared to contain a significant fraction of organic carbon (not revealed during XRD analyses), based on the formation of an opaque black solution after addition of 5 mL concentrated nitric acid to approximately 1 gram of a powdered pyrite sample. This phenomenon was also observed by Lord [26]. Based on the method of Huerta-Diaz and Morse [27], these samples were treated with concentrated H_2_SO_4 _for approximately 10 minutes and rinsed with ultrapure water, followed by ethanol. This appeared to eliminate the organic carbon.

### Dissolution Experiments

The dissolution behavior of pyrite material that had undergone dry sieving was compared to the dissolution of those that had been wet sieved. Prior to experimentation, all pyrite samples were treated to remove any surface iron oxides or iron sulfates that could have been produced when the samples were exposed to the atmosphere. This procedure, a modified version of a method used by Paschka and Dzombak [21], involved boiling 7–8 g of pyrite in 50 ml concentrated HCl for approximately 10 minutes. The sample was rinsed with boiling concentrated HCl at least twice, then rinsed with 25 ml deionized water, followed by a boiling acetone rinse using a vacuum filter. The acetone rinse was repeated at least 3 times. The sample was dried in the oven at 105°C for about 10 minutes and stored in a desiccator. Specific surface area measurements were conducted prior to the cleaning procedure.

Dissolution experiments were carried out in a batch reactor under tightly controlled conditions: pH = 3 ± 0.05, a constant temperature of 25 ± 0.01°C, fixed dissolved oxygen (8–11 ppm), and electrolyte solution of 0.01 M NaCl initial ionic strength [21]. A precise 5.355 ± 0.005 g aliquot of cleaned pyrite was added to 1.5 L of deionized water in a stirred, jacketed glass vessel with a lid having sealed ports for insertion of reagents and withdrawal of samples from the reactor. During the experiments, the reactor was covered with aluminum foil to exclude light. pH was maintained through the addition of HCl or NaOH via acid/base pumps and a pH-stat. Pyrite dissolution was monitored by measuring total dissolved iron. Five milliliters of sample were collected periodically over an 8 hour time period, and then filtered through a 0.45 μm disposable filter into a 20 ml polyethylene scintillation vial containing 5 ml 10% HNO_3 _for sulfur and iron analysis. Iron and sulfur concentrations were measured using ICP-AES, with replicate measurements of Fe by flame and graphite furnace AA.

### Analytical Methods

Specific surface area of each sample was measured by the nitrogen adsorption multipoint BET method with a Quantosorb instrument (Quanta Chrome, Boynton Beach, Florida). The accuracy of the instrument was verified by measurements on alumina and black carbon standards of known surface area. Particle surfaces were examined pre- and post- cleaning using a Philips XL-30 FEG field emission scanning electron microscope (Almelo, Netherlands). Sulfur and iron concentrations were measured on a SpectroFlame EOP ICP-AES (Kleve, Germany) using EPA Method SW 846 [28]. Accuracy of measurements are within ± 5% of true values. Replicate analyses of total dissolved iron were measured using a GBC 908AA atomic absorption spectrometer (GBC Scientific Equipment, Hampshire, IL). Instrument calibration was carried out using a suite of different concentrations of iron standard solution (Fisher Scientific) in 5% nitric acid matrix. All the aqueous samples were preserved in 5% nitric acid matrix before ICP-AES and AA measurements.

## Results

**Figure 2:**
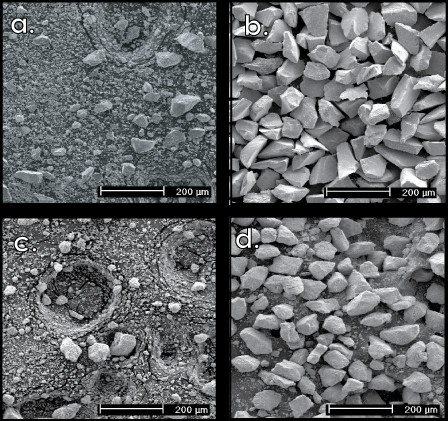
**Comparison of results obtained using the wet sieving technique and the dry sieving technique**. The wet sieving technique was successful in eliminating the aggregation of smaller size particles, achieving a narrow range of particle sizes for all samples, and removing adhering particles from the pyrite surface. **a**) Dry sieved, 63–75 μm, hydrothermal pyrite sample, HY-001, 63–75 μm, and **b**) HY-001, wet sieved, target size fraction 63–75 μm. **c**) Dry sieved, 44–75 μm, sedimentary pyrite sample, SED-002, and d) SED-002, wet sieved, target size fraction 44–75 μm.

**Figure 3:**
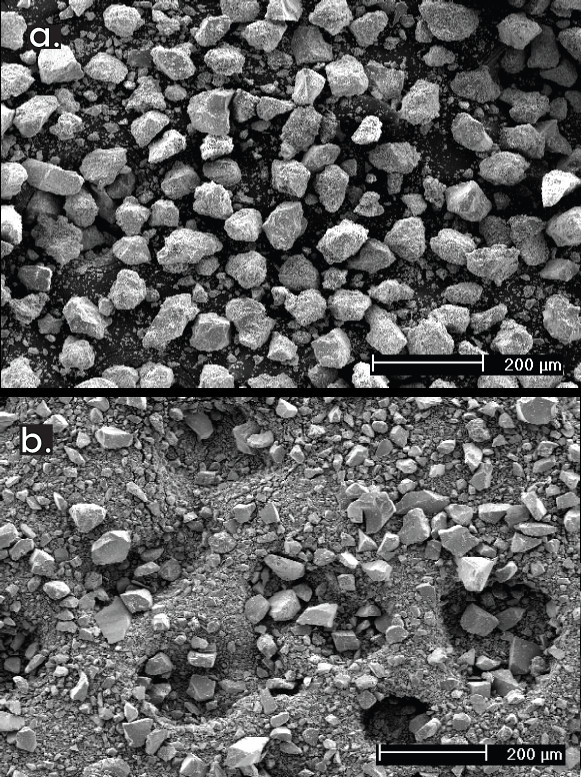
**Dry sieved samples after cleaning steps**. **a**) Sedimentary pyrite sample SED-003 showed some improvement after the tetrabromoethane cleaning procedure, although particles smaller than the finest sieve size (44 μm) clearly still remain. **b**) Hydrothermal pyrite HY-002 showed little improvement after ultrasonication cleaning procedure. See text for details of procedures.

SEM analyses of dry sieved samples indicated the presence of significant numbers of particles smaller than the desired range, *i.e*., <44 μm (Fig. 2a, c). In contrast, the wet sieving preparation method was successful at concentrating the intended particle size range and cleaning the surfaces (Fig. 2b, d). The addition of the tetrabromoethane cleaning step to the dry sieved samples reduced the number of <44 μm particles, but still left substantial numbers of fine particles (Fig. 3a). The ultrasonicating cleaning procedure was also largely unsuccessful in removing finer pyrite particles in the dry sieving method, based on SEM observation (Fig. 3b).

BET surface area measurements on dry sieved samples (63–75 μm) yielded a range from 0.2 to 3.1 m^2^/g, with SED-003>HY-001>SED-001>SED-002 (Table 3). Surface area measurements for wet sieved samples (44–75 μm) ranged from 0.2 to 5.4 m^2^/g, with SED-002>SED-001>SED-003>HY-001. The BET surface area of the dry sieved samples was surprisingly low, given the large number of fine particles observed by SEM. We note, however, that the BET method has a relatively high uncertainty at low surface area values. Further work needs to be undertaken to more fully address why the wet sieved material shows a larger apparent range of measured BET surface area values.

**Table 3:** Surface area and dissolution rates for pyrite samples after preparing material using the dry and wet sieving technique.

	Dry Sieving Preparation 63 – 75 μm		Wet Sieving Preparation 45 – 75 μm	
**Sample ID**	Initial Dissolution Rate *μg of Fe/min*	Surface Area *m*^*2*^/*g*	Initial Dissolution Rate *μg of Fe/min*	Surface Area *m*^*2*^/*g*

HY-001	34.1	1.9	3.8	0.22
HY-002	*na*	*na*	*na*	*na*
SED-001	70.4	0.90	21.1	2.8
SED-002	82.3	0.20	23.4	5.4
SED-003	81.8	3.1	0.02	0.42

Initial dissolution rates were from the dissolved iron concentration at 60 minutes. Based on measurements of black carbon and alumina standard materials, the estimated maximum error for surface area measurements is ± 0.6 m^2^/g.

Results of the dissolution experiments are reported in Table 3. Initial dissolution rates (Table 3) were calculated using iron concentrations measured one hour into the experiment. Dissolution rates calculated for dry sieved pyrite samples were highest for sedimentary samples and lowest for hydrothermal samples, with SED-002>SED-003>SED-001>HY-001. Dissolution rates obtained for hydrothermal samples yielded the lowest rates, regardless of whether they were wet or dry sieved. The highest dissolution rates were obtained from sedimentary samples that had been prepared using the dry sieve technique.

**Figure 4:**
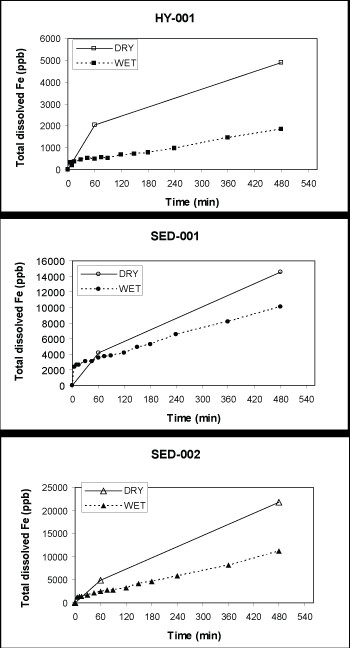
**Dissolution results for pyrite samples**. Dissolution results for three pyrite samples (one hydrothermal and two sedimentary) following preparation by dry and wet sieving. Samples were initially ground using a mixer mill. Dry sieved samples show much higher dissolution rates than samples that were wet sieved.

For wet sieved pyrites, the ranking of relative dissolution rates was similar to that of the dry sieved sedimentary pyrites, with SED-001>SED-002>SED-003>HY-001>HY-002. However, samples that were dry sieved achieved far higher absolute dissolution rates than those that were wet sieved (Fig. 4), even though the dry sieved samples were nominally sieved to a narrower particle size range (63–75 μm *vs*. 44–75 μm for wet sieved samples).

**Figure 5:**
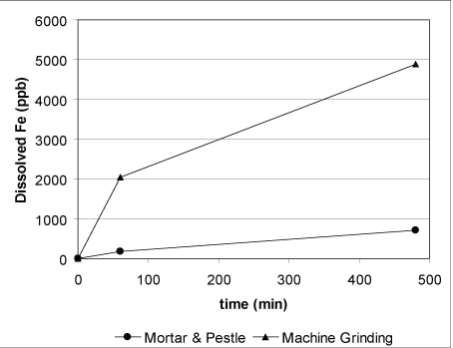
Comparison of cumulative iron concentration as a function of time for 63–75 μm splits of pyrite HY-001 produced by different grinding methods: hand grinding using an agate mortar and pestle vs. machine grinding using a mixer mill. In both cases, the ground samples were wet sieved.

A comparison of grinding techniques for sample HY-001 indicate that the rate of pyrite dissolution is higher for the sample powdered with the mixer mill than for the one prepared with the mortar and pestle (Fig. 5). SEM results suggest that machine grinding yielded a greater portion of grains skewed toward the lower end of the sieved size range, thus resulting in more exposed surface area and a higher dissolution rate.

## Discussion

### Dry sieving vs. wet sieving of crushed pyrite

Early efforts using the dry sieving technique to achieve a narrow range of fine particles yielded poor results; dry sieving resulted in a wide range of sizes from very fine particles adhering to the surface of individual grains to smaller particles (<44 μm) scattered throughout the larger matrix. Initially we attributed the aggregation of smaller sized particles to electrostatic charges being induced across the nylon mesh material, thus prohibiting movement through the sieve mesh, while the samples were sieved using the sieve shaker. However, changing from the polypropylene sieves to brass sieves did not improve the yield. The addition of tetrabromoethane to the sieving procedure helped marginally when the tetrabromoethane followed dry sieving, as the tetrabromoethane tended to clear the particle surfaces of finer particles. However, the finer particles were not removed from the sample, and using tetrabromoethane prior to dry sieving had little to no effect on the distribution of particles in the final sample.

Several workers ultrasonicated their respective samples in aqueous suspensions after dry sieving to remove fine particles from the pyrite surface [14,17,23]. However, this is not an effective particle separation method for fine grained (<100 μm) samples. Particles within our range of interest (45–75 μm) tend to remain suspended in the solution after ultrasonication. Decantation removes these particles along with other fine particles, ultimately biasing the method to retain larger sized particles within the collected size range.

The wet sieving technique was significantly more successful in producing a uniform distribution of particles in the size range of interest and is an effective method to produce a uniform, fine, and restricted pyrite particle size range for experiments. In addition, this technique does not require a post-processing cleanup step to remove adhering particles, as the particles are removed during the wet sieving procedure.

### Application to pyrite dissolution experiments

Results from the dissolution experiments indicate that pyrite preparation methods can affect the rate of dissolution significantly (Fig. 4). Pyrite powder prepared by dry sieving exhibited the highest surface area measurements and dissolution rates of all samples. Examination by SEM indicates that these results are likely caused by the presence of particles finer than 63 μm that adhere to larger particles even after separation with tetrabromoethane, and regardless of the type of sieve used.

## Conclusion

There is clearly a need to standardize sample preparation techniques to allow accurate comparisons of pyrite dissolution experiments under diverse conditions. We have developed a wet sieving procedure using vacuum filtration techniques to obtain fine (<100 μm) particle size fractions of pyrite for use in geochemical experiments. Compared to traditional methodologies, this procedure is far more successful at acquiring a narrow range (45–75 μm) of pyrite particles, as reflected in batch dissolution experiments and SEM analysis. This method uses readily available materials and equipment, and has potential application to other minerals as well.
